# Methionyl-tRNA formyltransferase utilizes 10-formyldihydrofolate as an alternative substrate and impacts antifolate drug action

**DOI:** 10.1099/mic.0.001297

**Published:** 2023-02-06

**Authors:** Shivjee Sah, Umesh Varshney

**Affiliations:** ^1^​ Department of Microbiology and Cell Biology, Indian Institute of Science, Bangalore, 560012, India; ^2^​ Jawaharlal Nehru Centre for Advanced Scientific Research, Bangalore, 560064, India

**Keywords:** 10-CHO-DHF, 10-CHO-THF, DHF, Fmt, folate pathway, one-carbon metabolic pathway, THF

## Abstract

Methionyl-tRNA formyltransferase (Fmt)-mediated formylation of Met-tRNA^fMet^ to fMet-tRNA^fMet^ is crucial for efficient initiation of translation in bacteria and the eukaryotic organelles. Folate dehydrogenase-cyclohydrolase (FolD), a bifunctional enzyme, carries out conversion of 5,10-methylene tetrahydrofolate (5,10-CH_2_-THF) to 10-formyl-THF (10-CHO-THF), a metabolite utilized by Fmt as a formyl group donor. In this study, using *in vivo* and *in vitro* approaches, we show that 10-CHO-DHF may also be utilized by Fmt as an alternative substrate (formyl group donor) to formylate Met-tRNA^fMet^. Dihydrofolate (DHF) formed as a by-product in the *in vitro* assay was verified by LC-MS/MS analysis. FolD-deficient mutants and Fmt over-expressing strains were more sensitive to trimethoprim (TMP) than the ∆*fmt* strain, suggesting that the domino effect of TMP leads to inhibition of protein synthesis and strain growth. Antifolate treatment to *Escherichia coli* showed a decrease in the reduced folate species (THF, 5,10-CH_2_-THF, 5-CH_3_-THF, 5,10-CH^+^-THF and 5-CHO-THF) and increase in the oxidized folate species (folic acid and DHF). In cells, 10-CHO-DHF and 10-CHO-folic acid were enriched in the stationary phase. This suggests that 10-CHO-DHF is a bioactive metabolite in the folate pathway for generating other folate intermediates and fMet-tRNA^fMet^.

## Introduction

**Fig. 1.**
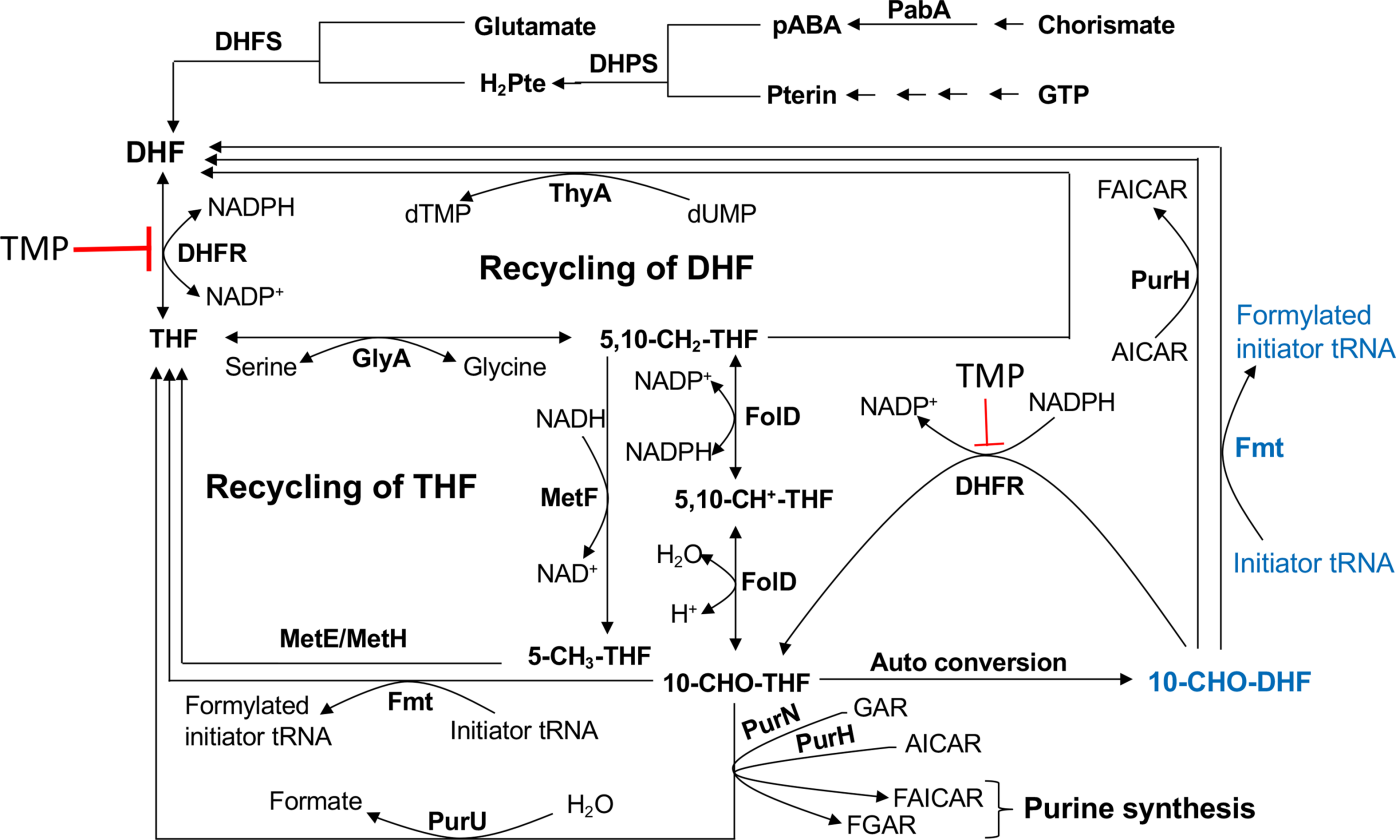
Schematic of the folate recycling pathway. The standard pathway includes dihydrofolate synthetase (DHFS), para-aminobenzoate synthase (PabA), dihydrofolate reductase (DHFR), serine hydroxymethyltransferase (GlyA), 5,10 methylenetetrahydrofolate dehydrogenase/cyclohydrolasse (FolD), 5,10-methylenetetrahydrofolate reductase (MetF), thymidylate synthase (ThyA), cobalamin-independent homocysteine transmethylase (MetE), cobalamin-dependent methionine synthase (MetH), phosphoribosylglycinamide formyltransferase (PurN) and AICAR transformylase/IMP cyclohydrolase (PurH), formyltetrahydrofolate deformylase (purU), 10-formyltetrahydrofolate: l-methionyl-tRNA^fMet^ *N*-formyltransferase (Fmt) is shown for the standard pathway (in black) and re-wired pathway (in blue).

The folate pathway (Fig. 1) is the key to the synthesis of glycine, methionine, thymidylate and purine nucleotides. The enzymes that catalyse inter-conversions of the pathway metabolites are highly conserved across the three domains of life [1–5]. Methylenetetrahydrofolate dehydrogenase-cyclohydrolase (FolD), which is mostly a bifunctional homodimeric protein in the one-carbon metabolic pathway, carries out sequential steps of conversion of 5,10-methylene-tetrahydrofolate (5,10-CH_2_-THF) to 5,10-methenyltetrahydrofolate (5,10-CH^+^-THF) by its dehydrogenase (EC 1.5.1.5) activity followed by the conversion of the latter to 10-formyltetrahydrofolate (10-CHO-THF) by its cyclohydrolase (EC 3.5.4.9) activity [6–8]. 10-CHO-THF, an important folate metabolite, is utilized by glycinamide ribonucleotide transformylase (PurN, EC 2.1.2.2) and bifunctional enzyme PurH, EC 2.1.2.3 [aminoimidazole carboxamide ribonucleotide (AICAR) transformylase and inosine 5′-phosphate (IMP) cyclohydrolase], in the purine nucleotide biosynthetic pathway [9, 10]. 10-CHO-THF is also utilized by UDP-4-amino-4-deoxy-l-arabinose formyltransferase (ArnA, EC 2.1.2.13) to formylate UDP-4-amino-4-deoxy-beta-l-arabinopyranose [11], and by formyltetrahydrofolate deformylase (PurU, EC 3.5.1.10) for the formation of formate under aerobic conditions. Thus, it is involved in homeostasis of the folate pool [12]. The availability of 10-CHO-THF is also vital for the formylation of the initiator tRNA (tRNA^fMet^) in bacteria and eukaryotic organelles by methionyl-tRNA formyltransferase (Fmt, EC 2.1.2.9) [13–15]. The formylation of the amino acid attached to the tRNA^fMet^ by Fmt was recently shown to be important in the fidelity of translation initiation [16, 17].

Dihydrofolate reductase (DHFR) catalyses the reduction of 7,8-dihydrofolate (DHF) to 5,6,7,8-tetrahydrofolate (THF), as well as of 10-formyldihydrofolate (10-CHO-DHF) to 10-formyltetrahydrofolate (10-CHO-THF). Thus, DHFR plays a central role in the maintenance of cellular pools of folate species [18–20]. DHFR is a well-known target of methotrexate and trimethoprim (TMP) for inhibiting growth of cancer cells and bacteria [21, 22]. PurH has also been studied extensively as a target of specific inhibitors for the development of anticancer therapeutics [23, 24]. The PurH enzymes reported from prokaryotes and eukaryotes utilize both 10-CHO-THF and 10-CHO-DHF [25]. Like DHFR and PurH, Fmt is present in both prokaryotes and the eukaryotic organelles. However, Fmt proteins have not been studied for the possibility of 10-CHO-DHF utilization and its physiological importance for antifolate drug action in any domains of life.

In this study, using a folate-deficient (∆*pabA*) *Escherichia coli *, we show that 10-CHO-DHF can be utilized by Fmt to formylate Met-tRNA^fMet^. Also, the *in vitro* reactions showed that the formyl group of 10-CHO-DHF was transferred to Met-tRNA^fMet^ by Fmt. *E. coli* strains mutated for FolD and/or those expressing Fmt were more sensitive to TMP than the ∆*fmt* strain. We show that 10-CHO-DHF is a bioactive metabolite in one-carbon metabolism.

## Methods

### Chemicals, plasmids, enzymes, *E. coli* strains and their growth

The (6*R*,*S*)-5,10-methylene-5,6,7,8-tetrahydrofolic acid, calcium salt (5,10-CH_2_-THF) and (6*S*)−5-formyl-5,6,7,8-tetrahydrofolic acid, calcium salt (5-CHO-THF) were sourced from Schircks Laboratories. Tetrahydrofolic acid was purchased from Sigma-Aldrich. Stock solution of 5,10-CH^+^-THF was prepared from 5-CHO-THF in acidic solution (0.1 M HCl and 0.1 M β-mercaptoethanol). The 10-CHO-THF was prepared from 5,10-CH^+^-THF in 50 mM Tris-Cl (pH 7.5) and 0.1 M β-mercaptoethanol under N_2_-sparged conditions on ice. The 10-CHO-DHF was prepared from 10-CHO-THF following air oxidation, as previously reported [26]. The radioisotopes were purchased from the Board of Radiation and Isotope Technology (India). *E. coli* strains, plasmids and enzymes are listed in Table 1. Bacteria were grown in Luria-Bertani broth (LB), LB-agar (1.8 % agar; Difco) or M9 minimal media (which includes 0.4 % glucose as a carbon source) containing 1 µg ml^−1^ thiamine at 37 °C. Ampicillin (Amp, 100 µg ml^−1^), kanamycin (Kan, 25 µg ml^−1^), chloramphenicol (Cm, 30 µg ml^−1^) and tetracycline (Tet, 7.5 µg ml^−1^) were used as needed.

**Table 1.** Description of *E. coli* strains, plasmids and oligos used in this study

* E. coli * strain/plasmid/enzymes	Genotype/details	Reference/source
BW25113	F^−^ DE(araD-araB)567 lacZ4787(del)::rrnB-3 LAM^-^ rph-1 DE(rhaD-rhaB)568 hsdR514	[44]
JW3323-1 (∆pabA::kan)	F^−^ DE(araD-araB)567 lacZ4787(del)::rrnB-3 LAM^−^ pabA768(del)::kan rph-1 DE(rhaD-rhaB)568 hsdR514	[44]
JW3970-1	F^−^ DE(araD-araB)567 lacZ4787(del)::rrnB-3 LAM^−^ purH777(del)::kan rph-1 DE(rhaD-rhaB)568 hsdR514	[44]
* E. coli* KL16	* E. coli* K-12 *thi1relA1 spoT1*	[45]
LH18 strain	(NM522 *thyA*∆*folA*::*kan*)	[46]
∆*pabA*	∆*pabA* strain of *E. coli* JW3323-1 was developed by removal of *kan* ^R^ marker from ∆*pabA::kan* strain with the help of pCP20 (32)	[47]
∆*pabA*∆*folA thyA*	Thymine-requiring derivative of ∆*pabA*∆*folA*::*kan*	This study
*folD*_wt_	KL16 derivative having *folD* locus. *Kan* ^R^ cassette is placed downstream of the *folD* gene for selection	[16]
*folD*_G122D_	KL16 derivative having mutated *folD* locus (G122D) transferred from the A48 suppressor. *Kan* ^R^ cassette is placed downstream of the *folD* gene for selection	[16]
*folD*_C58Y_	KL16 derivative having mutated *folD* locus (C58Y) transferred from B22 suppressor. *Kan* ^R^ cassette is placed downstream of the *folD* gene for selection	[16]
∆*fmt*	KL16 derivative having *fmt* gene replaced with *kan* ^R^ cassette	[29]
BL21(DE3)	* E. coli* strain suitable for high-level protein expression	Novagen
Rosetta (DE3) pLysS	* E. coli* strain suitable for high-level protein expression	Novagen
pET28b-*AtGGH2*	Folate gamma-glutamyl hydrolase from Arabidopsis was cloned in pET28b	[48]
p-FBT	The pLOI707HE vector containing the *slr*0642 cDNA of folate-biopterin transporter (FBT) family (Tet^R^)	[30]
met33	CTTCGGGTTATGAGCCCGACGAGCTA	[16]
pACDH	A low copy number plasmid with ACYC ori of replication, compatible with the ColE1 origin of replication (Tet^R^)	[49]
pACDH-*fmt*	*fmt* gene cloned in pACDH vector (Tet^R^)	[50]
p*metY*	pCAT_am1_ *metY* derivative with the WT *metY* gene but lacking the CAT reporter gene (generated by digestion with *Bam*HI and *Pst*I).	[51]
Fmt	Methionyl-tRNA formyltransferase (6XHis-tagged) from *E. coli* was purified using Ni-NTA column	Lab stock
MetRS	Methionyl-tRNA synthetase (6XHis-tagged) from *E. coli* was purified using Ni-NTA column	Lab stock
GlyA	Serine hydroxymethyltransferase (6XHis-tagged) from *E. coli* was purified using Ni-NTA column	Lab stock
FolD	Methylenetetrahydrofolate dehydrogenase-cyclohydrolase (6XHis-tagged) from *E. coli* was purified using Ni-NTA column	[6]
AtGGH2 (At1g78680)	Folate gamma-glutamyl hydrolase from Arabidopsis was expressed in BL21(DE3)/Rosetta (DE3) pLysS and purified using Ni-NTA column	Lab stock

### Growth curves

Overnight cultures of *E. coli* (three or four biological replicates) were grown in 2 ml LB at 37 °C with or without the desired antibiotic(s). The cultures were centrifuged and the pellets were resuspended in the same volume of M9 minimal medium. The cultures were diluted (10^3^-fold) in M9 minimal medium and 200 µl of the diluted cultures was taken into the wells of a honeycomb plate. The plate was placed in a Bioscreen C growth reader (Oy Growth). Culture growth was measured at OD_600_ at 1 h intervals and plotted using GraphPad Prism in which mean values with standard deviation (sd) were plotted against time.

### *In vivo* detection of formylation status of tRNA^fMet^ by Northern blotting

Total tRNAs from various strains were prepared under cold and acidic conditions to preserve the ester bond linking the amino acid to tRNA [27]. The aminoacyl-tRNA (Met-tRNA^fMet^) was deacylated with 10 mM CuSO_4_ in 100 mM Tris-HCl (pH 8.0) and both the formylaminoacyl- and the aminoacyl-forms of tRNA (fMet-tRNA^fMet^ and Met-tRNA^fMet^) were deacylated with 100 mM Tris-HCl (pH 9). The tRNAs were separated on acid urea PAGE and analysed by Northern blotting using a 5′-^32^P end-labelled DNA oligomer met33 (complementary to positions 25–39 of tRNA^fMet^) as described previously [27, 28].

### *In vitro* formylation assay

Total tRNA was prepared from the KL16∆*fmt* strain overexpressing initiator tRNA^fMet^ (p*metY*). The deacylated total tRNA preparations (10 µg) were incubated with 180 ng MetRS (* E. coli *) for 1 h in 10 µl aminoacylation buffer (50 mM HEPES buffer, pH 7.3, 25 mM KCl, 2 mM DTT, 10 mM MgCl_2_, 1 mM ATP, 0.1 % BSA and 2 mM methionine) as described previously [29]. The methionine charged tRNA^fMet^ was incubated with different folates (100 µM) along with Fmt (0.2 µg) for 10 min at room temperature in the aminoacylation buffer, mixed with equal volumes of acid urea dye (0.1 M sodium acetate pH 5.0, 10 mM Na_2_EDTA, 8 M urea, 0.05 % bromophenol blue and 0.05 % xylene cyanol FF), resolved on acid urea PAGE and analysed by Northern blotting using probes specific to tRNA^fMet^. The blots were exposed to a phosphor-imager screen for analysis on a Bio Image analyser (FLA5100; Fuji Film).

### Purification of total tRNA enriched for the initiator tRNA

Total tRNA was prepared from the KL16∆*fmt* strain overexpressing a plasmid-borne copy of *metY* (p*metY*), separated on native-PAGE and stained with ethidium bromide. The band corresponding to initiator tRNA (tRNA^fMet2^) was excised and cut into small pieces. The gel pieces were submerged in the buffer (50 mM Tris, pH 8, 5 mM Na_2_EDTA, 0.1 M LiCl and 0.05 % water-saturated phenol) and shaken gently overnight at room temperature. The supernatant was collected, and the process was repeated once more. The DEAE-cellulose column (DE52, 1 ml) was equilibrated with the buffer (50 mM Tris, pH 8, and 0.1 M LiCl). The supernatants were mixed and loaded onto the equilibrated DEAE-cellulose column. The column was washed with equilibration buffer (10 ml). The tRNA^fMet2^ was eluted with the elution buffer (50 mM Tris, pH 8, 5 mM Na_2_EDTA, 1 M LiCl). The fractions containing tRNA^fMet2^ were pooled and precipitated with ethanol.

### *In vitro* synthesis of 5,10-CH_2_-THF by using GlyA

The 5,10-CH_2_-THF was prepared enzymatically using GlyA. A typical modified reaction (100 µl) contained 1 mM THF, 20 mM β-mercaptoethanol, 10 mM serine, 50 mM Tris-HCl (pH 8.2) and purified GlyA (1 µg) [7]. The assay was incubated for 10 min at room temperature to synthesize 5,10-CH_2_-THF. Confirmation of 5,10-CH_2_-THF synthesis was done by an enzymatic assay using FolD (1 µg) and NADP^+^ (0.2 mM). The reaction mixture was incubated for 10 min at room temperature in the absence or presence of FolD. The reaction was stopped with 0.4 ml water containing 0.5 % HCl and incubated on ice for 10 min followed by centrifugation at 15 800 *g* for 5 min. The supernatant was used to measure absorbance of the product, 5,10-CH^+^-THF (λ_max_ = 350 nm, acidified pH) [8].

### Identification of Fmt reaction products by LC-MS/MS

Briefly, the purified deacylated initiator tRNA^fMet^ (100 µM) was incubated with 180 ng of MetRS for 1 h in aminoacylation buffer. The methionine-charged tRNA was incubated with 10-CHO-THF/10-CHO-DHF (25 µM) and Fmt (0.2 µg) for 10 min at room temperature in the aminoacylation buffer and followed by the addition of 0.1 M HCl and 0.1 M β-mercaptoethanol to stop the reaction. The acidified reaction mixture was centrifuged at 15 800 *g* to collect the supernatant for LC-MS analysis using an Impact HD (Bruker) ESI QTOF high-resolution mass spectrometer equipped with a Dionex Ultimate 3000 (Thermo) micro-LC. Aliquots (25 µl) were injected into the LC-MS device equipped with an Agilent poroshell 120(4.6×150 mm) SB-C18 column (2.7 µm particle size, column temperature 25 °C) using gradients of mobile phase A (0.1 % formic acid in water) and B (0.1 % formic acid in acetonitrile). The elution was carried out with 5 % B for 5 min, 5–18 % B for 13 min, 20–95 % B for 5 min and held at 95 % B for 10 min with a flow rate of 0.3 ml min^−1^. For equilibration of the column, a 30 min pre-run with initial gradient conditions was performed before each sample. The LC-MS was run in electrospray negative ionization mode. LC-MS data analysis software showed the intensity of extracted ion chromatograms of DHF (*m/z*, 442.1783), THF (*m/z*, 444.1600), 5,10-CH^+^-THF (*m/z*, 454.1485), 10-CHO-DHF (*m/z*, 470.1400) and 10-CHO-THF (*m/z*, 472.1580) in negative ionization mode. The identities of the folate species were verified by MS/MS data in CFM-ID or MetFrag, an online search tool.

### Analysis of folate metabolites using LC-MS

Overnight cultures of *E. coli* wild type (BW25113) and ∆*purH* strains were grown in 2 ml LB at 37 °C. The cultures were diluted (100-fold) in LB media (50–100 ml) and grown separately until the log phase or to stationary phase. Similarly, the ∆*purH* strain grown separately until the log phase and stationary phase was treated with TMP (10 µg ml^−1^) for 5 h. The cultures from wild type and ∆*purH* strains were harvested, and the pellets were washed twice with buffer [25 mM potassium phosphate (KPi), pH 7.2, and 0.2 M NaCl]. The pellets were resuspended in 0.1 M KPi buffer pH 7.2, 1 % ascorbic acid and 0.1 % β-mercaptoethanol (final pH of buffer ~6), heated at 95 °C for 10 min, vortexed and centrifuged. The lysates was treated with folate gamma-glutamyl hydrolase (AtGGH2, 10 µg) for 45 min at 37 °C to make the mono-glutamylated form of folate species, which was filtered through a 3 kDa cut-off centricon and the flow-through was injected into the LC-MS device equipped with a C18, 150×4.6 mm, 3 µm reversed-phase column as described [4]. The extracted ion chromatograms of folic acid (*m/z*, 440.1324), DHF (*m/z*, 442.1783), THF (*m/z*, 444.1600), 10-CHO-folic acid (*m/z*, 468.1272), 10-CHO-DHF (*m/z*, 470.1400), 10-CHO-THF/5-CHO-THF (*m/z*, 472.1580), 5,10-CH^+^-THF (*m/z*, 454.1485), 5,10-CH_2_-THF (*m/z*, 456.1636) and 5-CH_3_-THF (*m/z*, 458.1793) in the negative ionization mode were verified by MS/MS analysis in CFM-ID or MetFrag, an online search tool.

## Results

### Engineering 10-CHO-DHF uptake in *E. coli* complements folate homeostasis and formylation of tRNA^fMet^

**Fig. 2.**
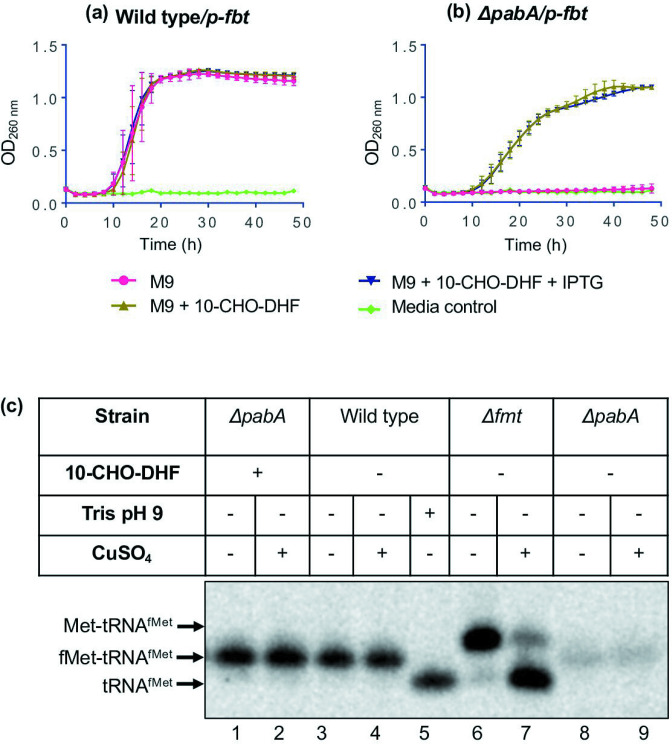
Uptake of 10-CHO-DHF improves folate homeostasis and formylation of tRNA^fMet^. (**a**) *E. coli* wild type (BW25113) strains harbouring a plasmid-borne gene encoding a folate transporter (p-*fbt*, Tet^R^) were grown in M9 media with or without supplementation of 10-CHO-DHF. The wild type strain grew in M9 medium. (**b**) The ∆*pabA* strain grew when supplemented with 10-CHO-DHF (100 µM) or/and IPTG (0.5 mM). (**c**) Total tRNA was prepared from *E. coli* wild type, ∆*pabA* and ∆*fmt* strains under acidic conditions. Uncharged (tRNA^fMet^), aminoacylated (Met-tRNA^fMet^) and formylated (fMet-tRNA^fMet^) forms of initiator tRNAs were separated on acid urea PAGE and analysed by Northern blotting with initiator-tRNA-specific probe (met33). The aminoacylated (Met-tRNA^fMet^) and formylated (fMet-tRNA^fMet^) forms of the initiator tRNA are deacylated by Tris-HCl, pH 9. However, only the aminoacylated form of these (Met-tRNA^fMet^) is deacylated with CuSO_4_ in the presence Tris-HCl, pH 8. The *Δfmt* strain is a control in which the initiator-tRNA is only methionylated but not formylated.

DHFR and PurH can utilize 10-CHO-DHF as substrate (*in vitro* and *in vivo*) to form 10-CHO-THF and DHF, respectively [18]. Thus, to investigate for 10-CHO-DHF utilization by Fmt *in vivo*, we decided to make use of a plasmid-borne gene encoding a folate transporter (on p-FBT, Tet^R^) to enable *E. coli* to obtain folates from the medium [30]. We chose an *E. coli* ∆*pabA* strain lacking *p*-amino benzoic acid (pABA), an essential metabolite for the biosynthesis of folates (Fig. 1). Expectedly, while the wild type strain grew, the ∆*pabA* strain did not grow in M9 media (Fig. 2a, b). However, its (∆*pabA*) growth was rescued when the medium was supplemented with 10-CHO-DHF (Figs 2b and S1, available in the online version of this article). Addition of IPTG along with 10-CHO-DHF to the ∆*pabA* strain did not make a difference under the conditions used (Fig. 2b). Further, we investigated whether 10-CHO-DHF can be utilized by folate-deficient bacteria (∆*pabA* strain) for formylation of initiator tRNA^fMet^. It was observed that the ∆*pabA* strain supplemented with 10-CHO-DHF showed accumulation of the formylated initiator tRNA^fMet^ as did the wild type *E. coli* (Fig. 2c). Note that a low level of formylated tRNA^fMet^ is also seen in the ∆*pabA* strain not supplemented with 10-CHO-DHF. This is most probably because the strain was not depleted for the residual levels of the folates (to enable accumulation of some cell mass). To pursue further the utilization of 10-CHO-DHF, we used a thymine-requiring (*thyA*) derivative of the ∆*pabA* ∆*folA::kan* strain. The strain grew better when the medium was supplemented with 10-CHO-DHF (Fig. S1B).

### Formylation of initiator tRNA^fMet^ with Fmt using different folate species

**Fig. 3.**
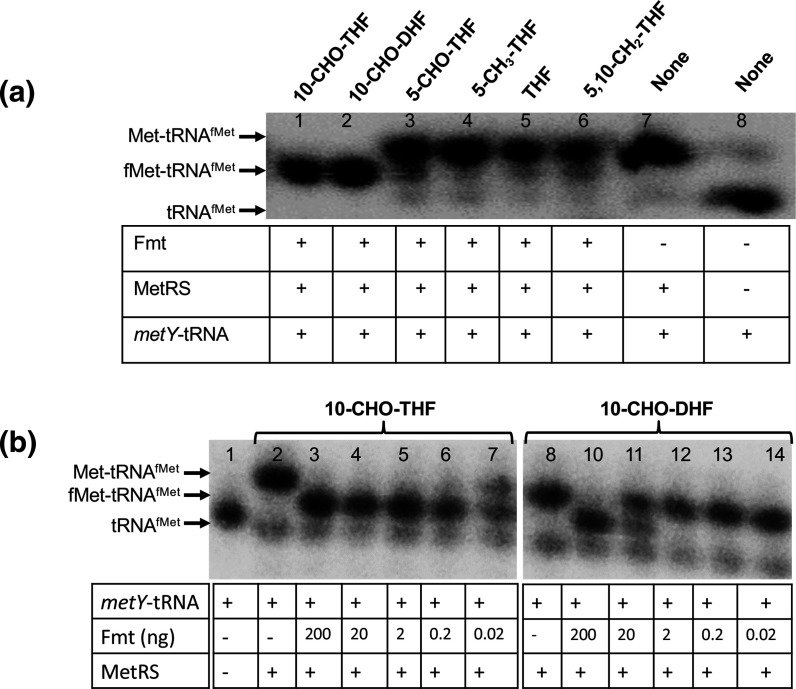
Formylation of Met-tRNA^fMet^ with 10-CHO-THF and 10-CHO-DHF by Fmt. Total deacylated tRNA (tRNA^fMet^) was converted into methionyl initiator tRNA^fMet^ (Met-tRNA^fMet^) with MetRS. Uncharged (tRNA^fMet^), aminoacylated (Met-tRNA^fMet^) and formylated (fMet-tRNA^fMet^) forms of initiator tRNAs were separated on acid urea PAGE and analysed by Northern blotting with initiator-tRNA specific probe (met33). (**a**) Met-tRNA^fMet^ was incubated with 10-CHO-THF, 10-CHO-DHF, 5-CHO-THF, THF, 5-CH_3_-THF or 5,10-CH_2_-THF along with Fmt. Met-tRNA^fMet^ and tRNA^fMet^ are shown as controls (Lanes 7 and 8). Met-tRNA^fMet^ was not formylated with 5-CHO-THF, 5-CH_3_-THF, THF or 5,10-CH_2_-THF (Lanes 3–6). Met-tRNA^fMet^ was formylated with 10-CHO-THF and 10-CHO-DHF (Lanes 1 and 2). (**b**) Met-tRNA^fMet^ (5 µM) was incubated with 10-CHO-THF or 10-CHO-DHF at 25 µM along with varying dilutions of Fmt (200–0.02 ng) for 7 min at room temperature.

Fmt activities were measured for their formylation reactions of methionyl initiator tRNA^fMet^ (Met-tRNA^fMet^) with different folate species and analysed by Northern blotting using acid urea gels. The use of either 10-CHO-THF or 10-CHO-DHF showed quantitative formylation of Met-tRNA^fMet^ (Figs 3a and S2). The commercially available 5,10-CH_2_-THF (1 mM) also showed formylatation of Met-tRNA^fMet^ (Fig. S2). However, to see if 5,10-CH_2_-THF indeed served as a substrate for Fmt, we first synthesized 5,10-CH_2_-THF *in vitro* by employing GlyA enzyme and then used it in the Fmt reaction. The synthesis of 5,10-CH_2_-THF was confirmed by a coupled enzyme assay using FolD (Fig. S3). 5,10-CH_2_-THF was not used as a substrate by Fmt (as was the case with the other folate species such as 5-CHO-THF, THF and 5-CH_3_-THF). While both 10-CHO-THF and 10-CHO-DHF were used by Fmt to quantitatively formylate Met-tRNA^fMet^ (Fig. 3a, lanes 1 and 2), based on the range finding assays using enzyme dilutions, the efficiency of utilization of 10-CHO-DHF by Fmt was about 0.1–1 % of that of 10-CHO-THF (Fig. 3b, compare lanes 6 and 7 with lanes 10 and 11) (see also the Discussion).

### Identification of the products of Fmt reactions by LC-MS/MS

**Fig. 4.**
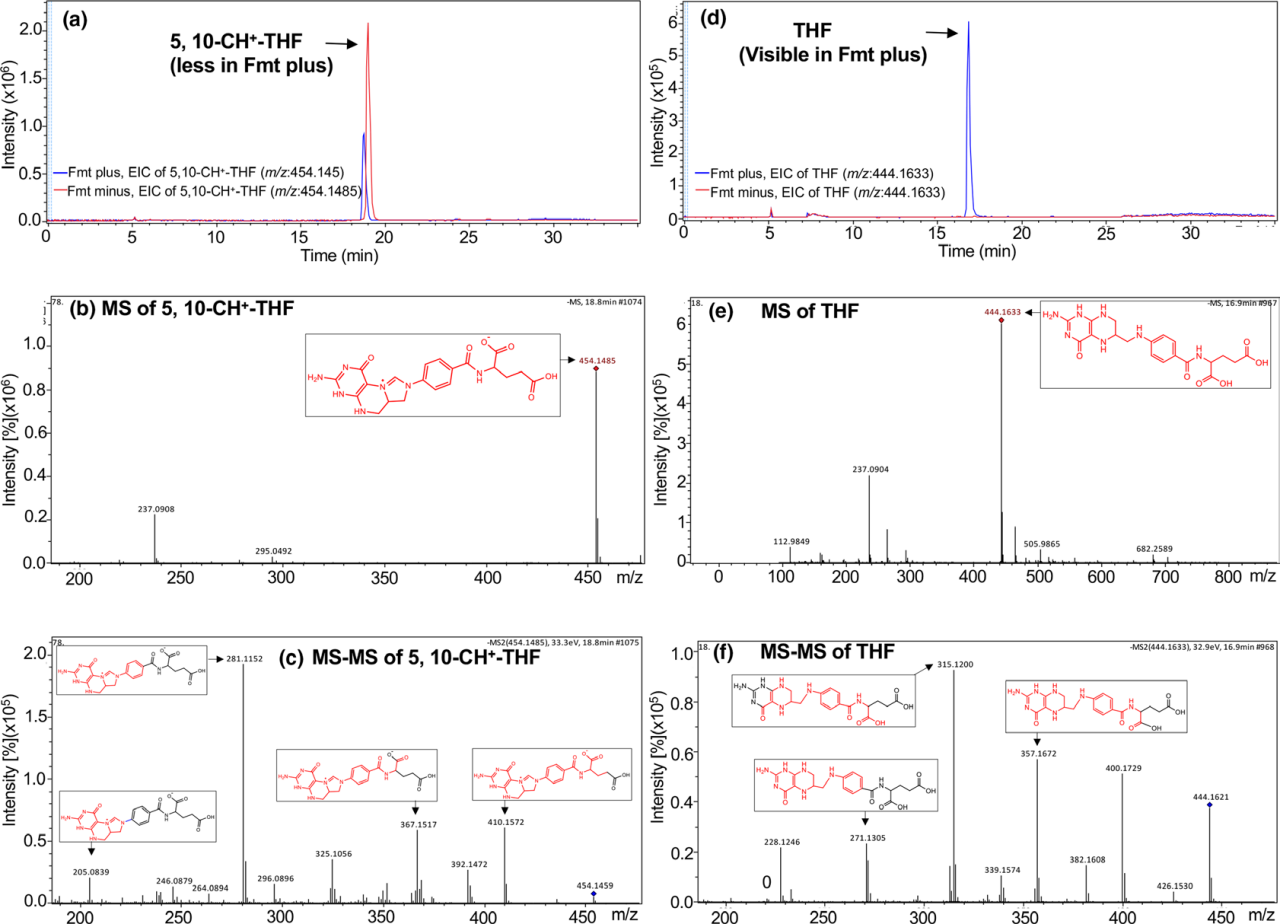
Identification of 10-CHO-THF as a substrate of Fmt by LC-MS/MS. The product THF formed in the reaction mixtures of Fmt enzyme when 10-CHO-THF used as a substrate was analysed by LC-MS/MS. (**a**) The EIC intensity of 5,10-CH^+^-THF (acidic 10-CHO-THF) in the reaction mixture decreased after addition of Fmt (blue line) in comparison to the one without Fmt (red line). (**b**) 5,10-CH^+^-THF was identified based on the MS peak at 454.1485 (*m*/*z*). (**c**) The MS ion of 5,10-CH^+^-THF (*m*/*z*=454.1485) was further fragmented into characteristic peaks by MS/MS analysis. (**d**) The EIC intensity of THF in the reaction mixture increased after addition of Fmt (blue line) in comparison to the one without Fmt (red line). (**e**) The product, THF, was identified based on the MS peak at 444.1633 (*m*/*z*). (**f**) The MS ion of THF (*m*/*z*=444.1633) was further fragmented into many peaks by MS/MS analysis. Structures of the relevant molecules are shown within the panels. The structural parts of the molecules corresponding to the fragmentation pattern (major *m/z* peaks) have been drawn in red (within the corresponding molecules). For further details see Data S1 and S2.

We observed that Fmt utilized both 10-CHO-THF and 10-CHO-DHF as substrates. To identify the products formed in Fmt reactions, LC-MS/MS analysis was performed within half an hour of the reaction. The Fmt reaction forms THF when 10-CHO-THF is used. However, in the assay, as the Fmt reaction containing 10-CHO-THF is stopped with HCl, the reaction mixture becomes acidic. Under these conditions, the 10-CHO-THF is converted to 5,10-CH^+^-THF. The EIC intensity of 5,10-CH^+^-THF in the reaction mixture decreased after addition of Fmt in comparison to the one without Fmt (Fig. 4a). The identity of 5,10-CH^+^-THF was verified with an MS peak at 454.1485 (*m*/*z*) (Fig. 4b). The ion was further verified by fragmenting it into the characteristic peaks by MS/MS (Fig. 4c, Data S1). Even under acidic conditions, the integrity of 10-CHO-THF was seen to some extent and the EIC of 10-CHO-THF (*m/z*, 472.1583) decreased upon addition of Fmt (Fig. S4A). The ion was further verified by MS and MS/MS analysis (Fig. S4B, C). Thus, the product of the Fmt reaction produced THF (*m/z*, 444.1632) which was verified by an increase in the intensity of its EIC (Fig. 4d, e). The fragmentation pattern of THF was visualized by MS/MS analyses (Fig. 4f and Data S2). However, when the Fmt reaction, after acidification, was kept for 4 h, it resulted in degradation of THF to 4-aminobenzoyl-l-glutamic acid (*m/z*, 265.0851) which was also verified by MS and MS/MS analyses (Fig. S5A–C).

**Fig. 5.**
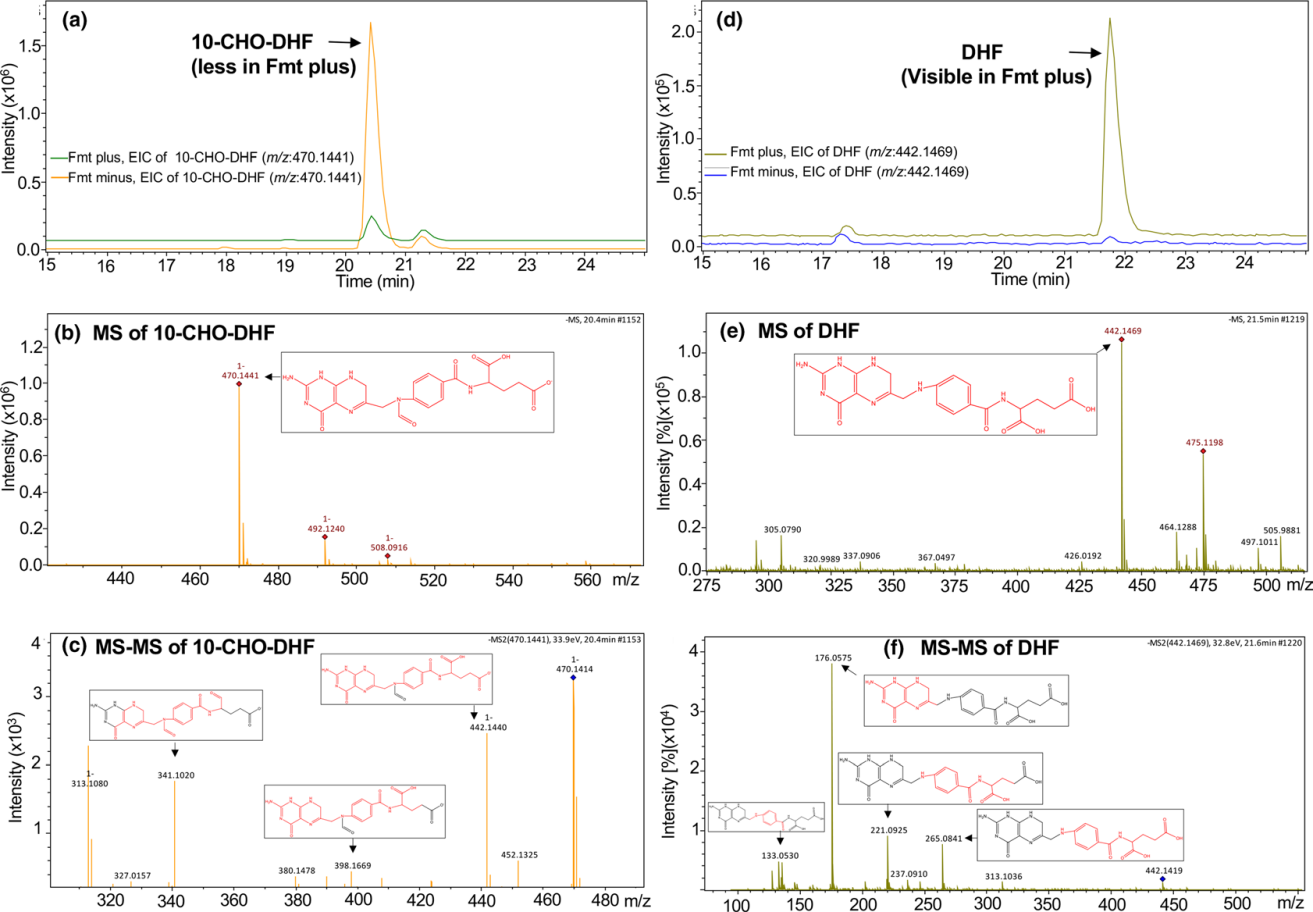
Identification of 10-CHO-DHF as the substrate of Fmt by LC-MS/MS: the product DHF formed in the reaction mixtures of Fmt enzyme when 10-CHO-DHF used as a substrate was analysed by LC-MS/MS. (**a**) The EIC intensity of 10-CHO-DHF in the reaction mixture decreased after addition of Fmt (green line) in comparison to the one without Fmt (orange line). (**b**) The substrate, 10-CHO-DHF, was identified based on the MS peak at 470.1441 (*m*/*z*). (**c**) The MS ion of 10-CHO-DHF (*m*/*z*=470.1441) was further fragmented into characteristic peaks by MS/MS analysis. (**d**) The EIC intensity of DHF in the reaction mixture increased after addition of Fmt (light green line) in comparison to the one without Fmt (blue line). (**e**) The product, DHF, was identified based on the MS peak at 442.1469 (*m*/*z*). (**f**) The MS ion of DHF (*m*/*z*=442.1469) was further fragmented into characteristic peaks by MS/MS analysis. Structures of the relevant molecules are shown within the panels. The structural parts of the molecules corresponding to the fragmentation pattern (major *m/z* peaks) have been drawn in red (within the corresponding molecules). For further details see Data S3 and S4.

Formation of DHF was expected in the Fmt reaction with 10-CHO-DHF as substrate. LC-MS/MS analysis showed that the EIC intensity of 10-CHO-DHF in the reaction mixture decreased after addition of Fmt in comparison to the reaction without Fmt (Fig. 5a). The identity of 10-CHO-DHF was verified by MS (*m*/*z,* 470.1441) and the MS/MS fragmentation pattern (Fig. 5b, c and Data S3). The product of the Fmt reaction was DHF (*m/z*, 442.1469), which was verified by an increase in the intensity of EIC (Fig. 5d, e). The fragmentation pattern of DHF was visualized by MS/MS analyses (Fig. 5f and Data S4).

### Fmt overexpression causes toxicity and sensitivity to TMP

**Fig. 6.**
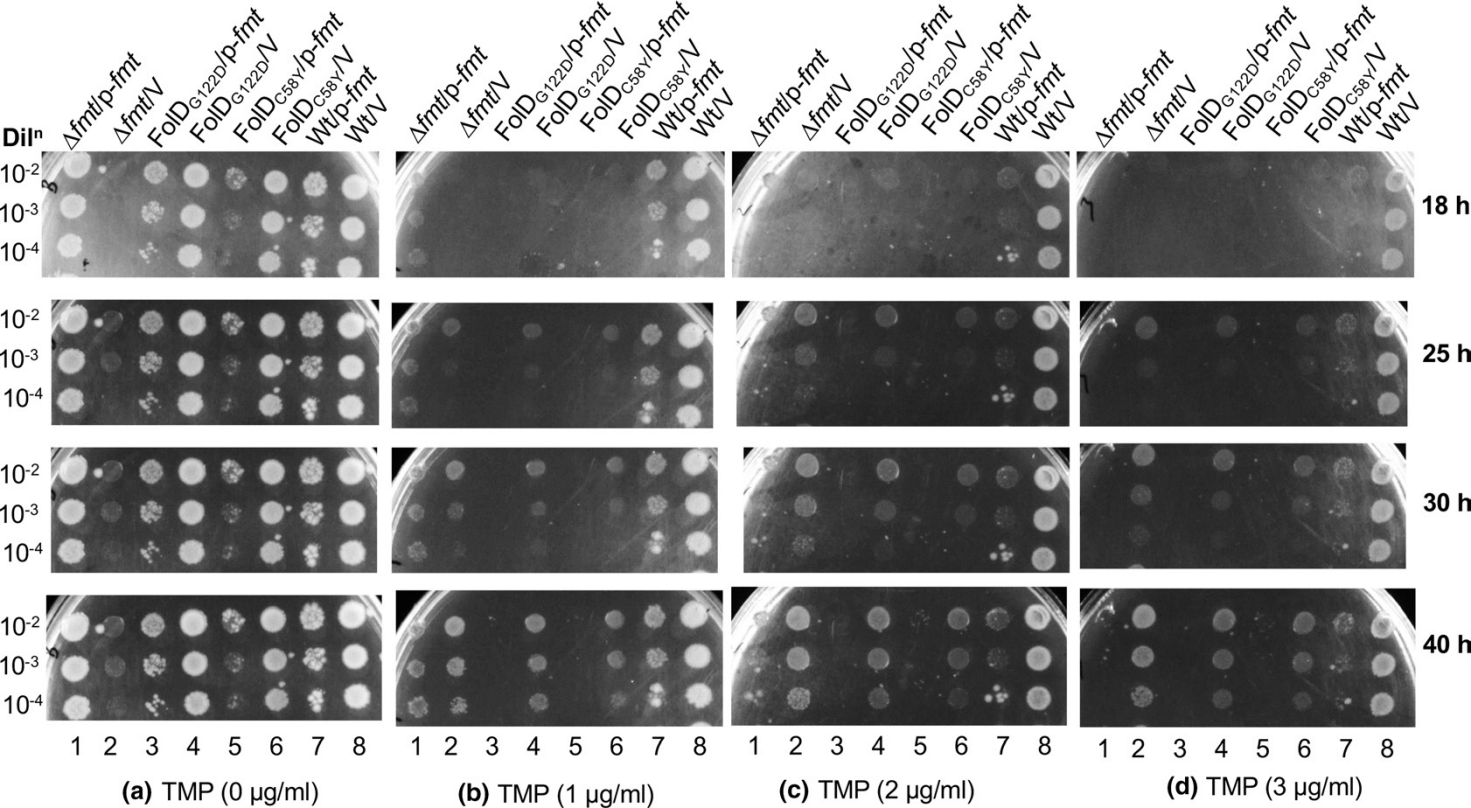
Fmt overexpression causes toxicity and sensitivity to trimethoprim. *E. coli* wild type, ∆*fmt* and FolD mutant strains harbouring plasmid alone (pACDH) or plasmid-borne *fmt* (*p-fmt*, Tet^R^) were spotted on LB agar with or without TMP and incubated for 18, 25, 30 and 40 h at 37 °C. (**a**) (without TMP) Growth of wild type and the *folD* mutant strains were retarded when Fmt was over-expressed (compare lane 7 with 8; lane 5 with 6; and 3 with 4). However, growth of the ∆*fmt* strain was enhanced when Fmt was over-expressed (compare lane 2 with 1). (**b–d**) In the presence of TMP, growth of wild type, ∆*fmt* and the *folD* mutant strains (harbouring vector alone, V) was retarded when Fmt was over-expressed (p-*fmt*). However, the ∆*fmt* strain showed slight resistance to TMP (lane 2 in panels b–d). Details of the strains are ∆*fmt*/V: ∆*fmt*/pACDH, ∆*fmt*/p-*fmt*: ∆*fmt*/pACDH*-fmt*, Wt/V: *folD*_wt_/pACDH, Wt/p-*fmt: folD*_wt_/pACDH-*fmt*, *folD*_C58Y_/V: *folD*_C58Y_/pACDH, *folD*_C58Y_/p-*fmt: folD*_C58Y_/pACDH-*fmt*, *folD*_G122D_/V: *folD*_G122D_/pACDH and *folD*_G122D_/p-*fmt: folD*_G122D_/pACDH-*fmt*.

**Fig. 7.**
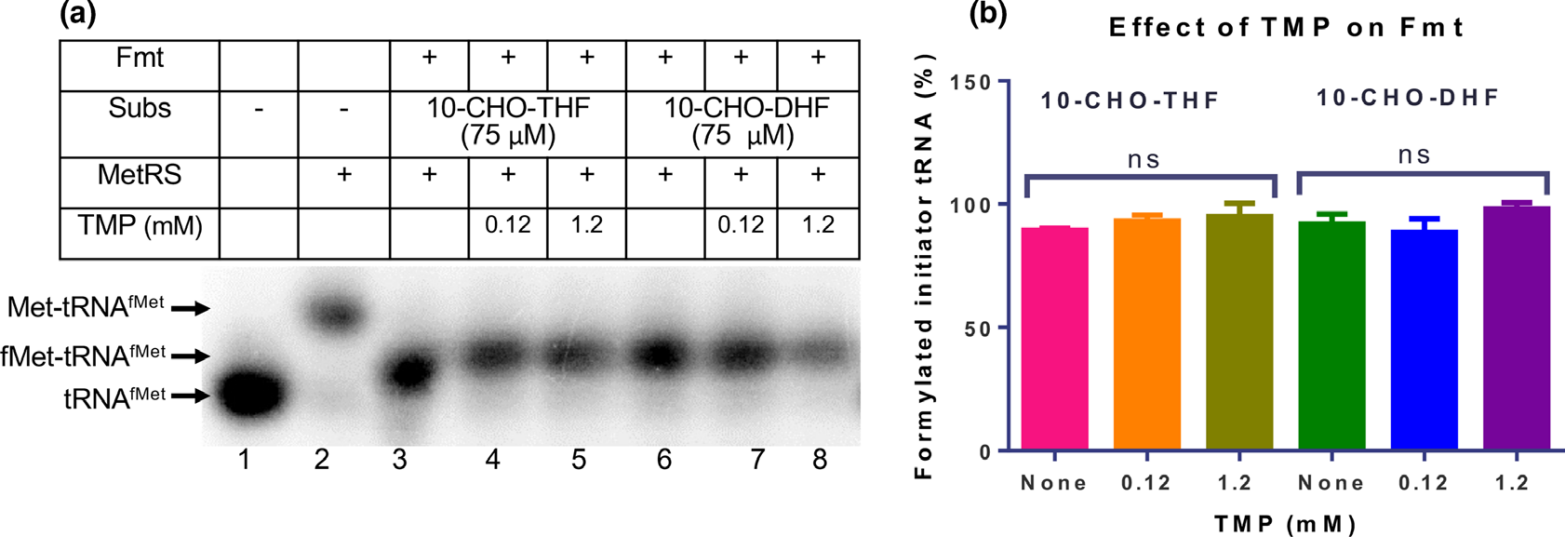
Formylation of Met-tRNA^fMet^ by Fmt is unchanged with TMP. An *in vitro* Fmt assay was performed with or without TMP in the presence of 10-CHO-THF or 10-CHO-DHF. (**a**) Total deacylated tRNA (tRNA^fMet^) was converted into methionyl initiator tRNA^fMet^ (Met-tRNA^fMet^) with MetRS. Met-tRNA^fMet^ was incubated with 10-CHO-THF or 10-CHO-DHF along with Fmt. Uncharged (tRNA^fMet^), aminoacylated (Met-tRNA^fMet^) and formylated (fMet-tRNA^fMet^) forms of initiator tRNA were separated on acid urea PAGE and analysed by Northern blotting with initiator-tRNA-specific probe (met33). tRNA^fMet^ and Met-tRNA^fMet^ are shown as a control (Lanes 1 and 2). Met-tRNA^fMet^ was formylated with 10-CHO-THF or 10-CHO-DHF by Fmt (Lanes 3 and 6). (**b**) The formylation of Met-tRNA^fMet^ (fMet-tRNA^fMet^) by Fmt was quantified from the Northern blots. The formylation of Met-tRNA^fMet^ with 10-CHO-THF or 10-CHO-DHF by Fmt in the presence of TMP was unchanged. Error bars represent sd of three independent experiments. The *P* value calculated with an unpaired *t*-test was nonsignificant (ns).

DHFR is known to utilize both DHF and 10-CHO-DHF as substrates [18] and is inhibited by TMP [21, 22]. Since Fmt is also noted to utilize 10-CHO-DHF as an alternative substrate, we checked the connection between Fmt and TMP. Further, we recently showed that *folD* mutants result in decreased production of 10-CHO-THF, which in turn impacts the rates of formylation of initiator tRNA^fMet^ [16]. Hence, in this analysis, we included the *folD* mutants (C58Y and G122D). Growth of the wild type and the *folD* mutant strains were retarded when Fmt was over-expressed (compare the strains harbouring empty vector or *fmt* in Fig. 6a), suggesting that overexpression of Fmt is toxic to these cells. The FolD mutants were more sensitive to TMP (compare the strains in Fig. 6b–d over the different times of incubation of the plates). Interestingly, we observed that even though the ∆*fmt* strain was compromised in its growth (because of *fmt* deletion), based on its growth over the times of incubation of the plates having different TMP concentrations, it showed slight resistance to TMP (compare lane 2 of Fig. 6a with lane 2 of Fig. 6b–d), and the overexpression of Fmt in the strain (∆*fmt* strain) made it sensitive to TMP on LB agar (compare lane 1 of Fig. 6a, with lane 1 of Fig. 6b–d). However, the ∆*fmt* strain did not show better growth in LB or modified M9 media than the media containing TMP (Fig. S6A, B). When we performed an *in vitro* enzyme assay, it was seen that Fmt was not inhibited by TMP (Fig. 7), and the effect of TMP in an Fmt overexpression background must be indirect (see Discussion).

### Accumulation of 10-CHO-DHF in stationary phase

**Fig. 8.**
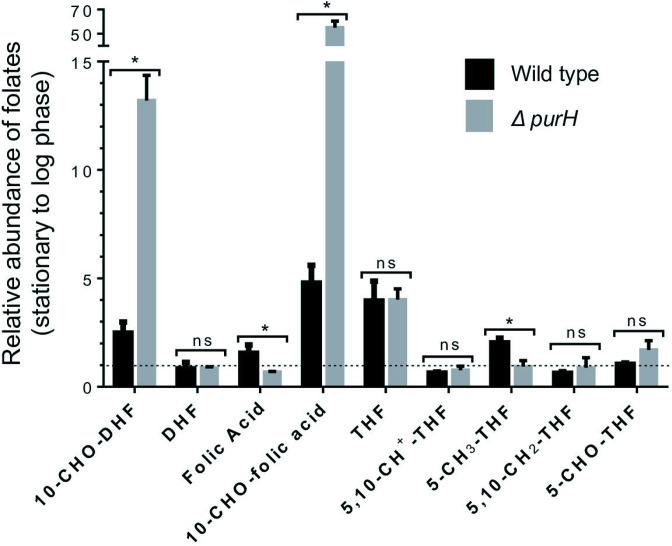
Enrichment of 10-CHO-DHF in the stationary phase. Folate metabolites were analysed from wild type (BW25113) and ∆*purH* (treated with TMP) strains by using LC-MS/MS and plotted as ratios of stationary phase to log phase. Error bars represent sd of three replicates. Statistical significance was determined with multiple *t*-tests with alpha=5 %. *P*=0.0001, 0.017, 0.0001 and 0.0056 for 10-CHO-DHF, folic acid, 10-CHO-folic acid and 5-CH_3_-THF, respectively. For the remainder, the *P* value was non-significant (ns).

While it is known that 10-CHO-DHF can be utilized by DHFR and PurH [18], it is not known if 10-CHO-DHF is also a natural metabolite in bacteria. Thus, we asked if treatment of the ∆*purH* strain (deleted for PurH) with TMP (to inhibit DHFR) would lead to accumulation of 10-CHO-DHF. We noted that in the ∆*purH* strain (compared to the wild type strain), the reduced folate species (THF, 5,10-CH_2_-THF, 5-CH_3_-THF, 5,10-CH^+^-THF and 5-CHO-THF) were decreased, and the oxidized folate species (folic acid and DHF) were increased (Figs S7 and S8). Levels of 10-CHO-folic acid and 10-CHO-DHF were decreased in the log phase grown ∆*purH* strain treated with TMP. However, levels of the same metabolites were increased in the stationary phase grown ∆*purH* strain treated with TMP (compare Figs S7 and S8). Levels of 10-CHO-DHF and 10-CHO-folic acid were higher in the stationary phase grown cells in comparison to the log phase grown cells (wild type). Levels of 10-CHO-DHF and 10-CHO-folic acid DHF were ~5-fold greater in the ∆*purH* strain than in the stationary phase grown wild type cells (Fig. 8).

## Discussion

The importance of the folate metabolic pathway has been extensively explored [1–3, 31]. The folate metabolite 10-CHO-THF is utilized by PurN and PurH in the purine nucleotide biosynthetic pathway and is vital for cell survival [9, 10, 32]. DHFR and PurH, which have also been extensively studied as targets of specific inhibitors for the development of anticancer therapeutics, have also been reported to use both the 10-CHO-THF and 10-CHO-DHF as formyl group donors [19, 20, 24, 25].

10-CHO-THF is also utilized by Fmt for formylation of Met-tRNA^fMet^ as a crucial step in translation initiation in bacteria and eukaryotic organelles [13–15]. Fmt has been well characterized from many organisms using its primary substrate 10-CHO-THF [17, 33–35]. In the present study, we investigated the potential utilization of 10-CHO-DHF by *E. coli* Fmt and showed that 10-CHO-DHF can be used as an alternative substrate. Additionally, at least under the conditions of Fmt sufficiency, Met-tRNA^fMet^ is quantitatively formylated using 10-CHO-DHF (Fig. 3a). Previous studies have shown that *E. coli* possesses about 100 molecules of Fmt per cell, and a decrease in Fmt levels even by 100-fold lead to only ~3-fold compromise in the overall formylation levels of Met-tRNA^fMet^ [29, 36, 37]. Thus, even though an *in vitro* range finding experiment (Fig. 3b) shows that Fmt uses 10-CHO-DHF at an efficiency of about 0.1–1 % of its canonical substrate, 10-CHO-THF, its use as an alternative substrate is relevant under *in vivo* conditions especially during the stationary phase where 10-CHO-DHF and 10-CHO-folic acid are enriched. Formylation of Met-tRNA^fMet^ was apparently seen also with the commercially available 5,10-CH_2_-THF (Fig. S2). However, with the freshly synthesized 5,10-CH_2_-THF, no detectable formylation of Met-tRNA^fMet^ was seen, suggesting unsuitability of the stored 5,10-CH_2_-THF for such analyses.

From the *in vivo* experiments, it was observed that the ∆*pabA* strain grew well in M9 media supplemented with 10-CHO-DHF, and it accumulated the formylated form (fMet-tRNA^fMet^) of the initiator tRNA^fMet^ (Fig. 2). However, under the limiting levels of 10-CHO-DHF, both the intensity of formylated Met-tRNA^fMet^ and the growth of the ∆*pabA* strain decreased (Fig. 2). These observations suggest that the utilization of 10-CHO-DHF is not only for formylation of Met-tRNA^fMet^ but also to generate other essential intermediary metabolites for growth.

It was observed that Fmt overexpression caused toxicity/sensitivity to TMP even in rich media (Fig. 6). Use of rich growth medium excludes the possibility of the limitation of purine nucleotides, thymidine and amino acids. However, even in enriched media, the deletion of FolD (which synthesizes 10-CHO-THF) was unsuccessful [7]. Thus, this observation may indicate the unstable nature of the folate metabolites, particularly 10-CHO-THF, used for formylation of Met-tRNA^fMet^ by Fmt. The formylation of Met-tRNA^fMet^ by Fmt is essential for normal growth of bacteria, and it favours initiation with fMet-tRNA^fMet^ by increasing its affinity to IF2 for its recruitment on the ribosome [13, 38]. Recently, we showed that a low level of cellular Fmt or 10-CHO-THF (due to compromised FolD) leads to a compromised efficiency of formylation of Met-tRNA^fMet^ [6, 16, 29]. TMP is well known to inhibit DHFR, which in turn leads to decreased production of folate, purines, thymidine, amino acids and *S*-adenosylmethionine (SAM) [28, 39–41], and overexpression of Fmt makes the strain more sensitive to TMP (Fig. 6). This also suggests implications of Fmt in TMP mediated toxicity. However, *in vitro* studies of Fmt activity assays suggested that TMP does not directly inhibit Fmt (Fig. 7). A probable explanation for TMP's sensitivity upon Fmt overexpression might be the limitation of 10-CHO-THF for other essential reactions, which is further enhanced by preferential utilization/sequestration of the available 10-CHO-THF by Fmt. Thus, TMP-mediated inhibition of DHFR leads to blockade of not only folate, purines, thymidine, amino acids and SAM but also of protein synthesis. The domino effect of the action of TMP was also reported wherein it blocks not only DHFR but also another critical enzyme of folate metabolism, folylpoly-gamma-glutamate synthetase (FP-Υ-GS) indirectly [41]. We found that the deletion of *fmt* (∆*fmt* strain) confers resistance to TMP, which signifies that the already limiting amount of 10-CHO-THF is not sequestered by Fmt and thus the ∆*fmt* strain becomes resistant to TMP on the LB-agar plate. However, growth of the ∆*fmt* strain is not resistant to TMP in the media under the shaking conditions of growth.

As expected, TMP-treated *E. coli* ∆*purH* grown in enriched media showed an overall increase in oxidized folates and a decrease in reduced folates compared with the wild type strain. A similar observation was made when TMP-treated wild type *E. coli* was grown in minimal media [41].

Among the folates, 10-CHO-DHF was not reported from *E. coli* grown to the log phase [41]. The levels of 10-CHO-DHF and 10-CHO-folic acid were higher in the stationary phase compared to cells grown to the log phase. The levels of same metabolites were >5-fold higher in the ∆*purH* strain treated with TMP than the wild type cells in the stationary phase. However, the expected precursor compounds (such as 5,10-CH^+^-THF, 5-CHO-THF/10-CHO-THF) of 10-CHO-DHF and 10-CHO-folic acid were >10-fold less in the ∆*purH* strain treated with TMP than the wild type cells in the stationary phase (Fig. S8). Similar to this study, the formation of 10-CHO-folic acid, a potent inhibitor of DHFR, in rat liver slices incubated with folic acid and methotrexate was observed, where reduction of folates has not been shown to occur [42], suggesting accumulation of 10-CHO-DHF and 10-CHO-folic acid from an unknown pathway. Possible spontaneous oxidation of 10-CHO-DHF would generate 10-CHO-folic acid [43]. It would be interesting to see if 10-CHO-folic acid could also be used by Fmt as a substrate. Nonetheless, in *E. coli *, both 10-CHO-DHF/10-CHO-folic acid might serve as bioactive metabolites that enter one-carbon metabolism and other intermediary pathways for cell survival. Moreover, these compounds might be involved in metabolic regulation by inhibiting DHFR and other enzymes, particularly during the stationary phase for sustained growth of the culture.

Together, our study supports a major role of Fmt in the utilization of 10-CHO-THF and 10-CHO-DHF for the formylation of Met-tRNA^fMet^ and generating THF and DHF via the recycling pathway in the one-carbon metabolic pathway (Fig. 1). In conclusion, *in vivo* and *in vitro* characterization of Fmt from *E. coli* has allowed for a better understanding of the domino effect of TMP and utilization of 10-CHO-DHF in the folate pathway for generating other folate intermediates for the synthesis of purine nucleotides, glycine, thymine, methionine and formylated Met-tRNA^fMet^.

## Supplementary Data

Supplementary material 1
