# Supplementary material for: Methionyl-tRNA formyltransferase utilizes 10-formyldihydrofolate as an alternative substrate and impacts antifolate drug action
Source: Microbiology (Reading). 2023 Feb 6;169(2):001297. doi: 10.1099/mic.0.001297 (PMC10197868; doi:10.1099/mic.0.001297)
Supplement: Supplementary material 1 [file mic-169-1297-s001.pdf]

SUPPLEMENTARY DATA

Methionyl-tRNA formyltransferase utilizes 10-formyldihydrofolate as an alternate substate and impacts antifolate drug action

Shivjee Sah[1] and Umesh Varshney[1,2*]

[1]Department of Microbiology and Cell Biology, Indian Institute of Science, Bangalore, 560012, India, and [2]Jawaharlal Nehru Centre for Advanced Scientific Research, Bangalore, 560064, India

[*]*Correspondence to*: Umesh Varshney, Phone: +918022932686, Fax: +918023602697, E-mail: varshney@iisc.ac.in ; uvarshney@gmail.com

Keywords: Folate pathway, one-carbon metabolic pathway, DHF, 10-CHO-DHF, THF, 10-CHO-THF, Fmt

FIGURES S1-S8 (Pages 2-7)

DATA S1-S4 (Pages 8-11)

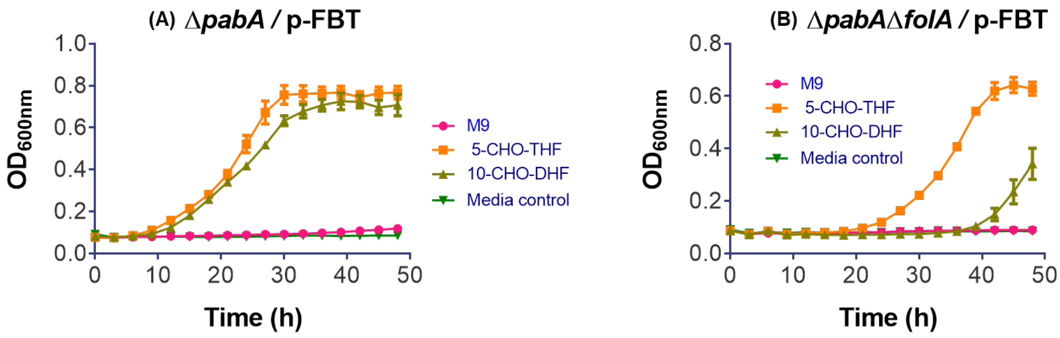

Figure S1. Uptake of 10-CHO-DHF by Δ*pabA* and Δ*pabA*Δ*folA* strains. *E. coli* Δ*pabA* and Δ*pabA*Δ*folA* strains harbouring plasmid borne gene encoding a folate transporter (p-FBT, Tet^R) were grown in M9 media with or without supplementation of 5-CHO-THF /10-CHO-DHF (50 µM each). **(A)** Δ*pabA*/p-FBT, **(B)** Thymine requiring (*thyA*) derivative of Δ*pabA*Δ*folA*/p-FBT strain grown in M9 media containing thymine 50 µg/ml.

FMT (ng)			800	160	30	800	160	30	800	160	30	800	160	30
Subs	-	-	10-CHO-THF (0.4 mM)			10-CHO-DHF (1 mM)			5,10-CH_2-THF (1 mM)			5-CH_3-THF (1 mM)		
MetRS		+	+	+	+	+	+	+	+	+	+	+	+	+
tRNA	+	+	+	+	+	+	+	+	+	+	+	+	+	+

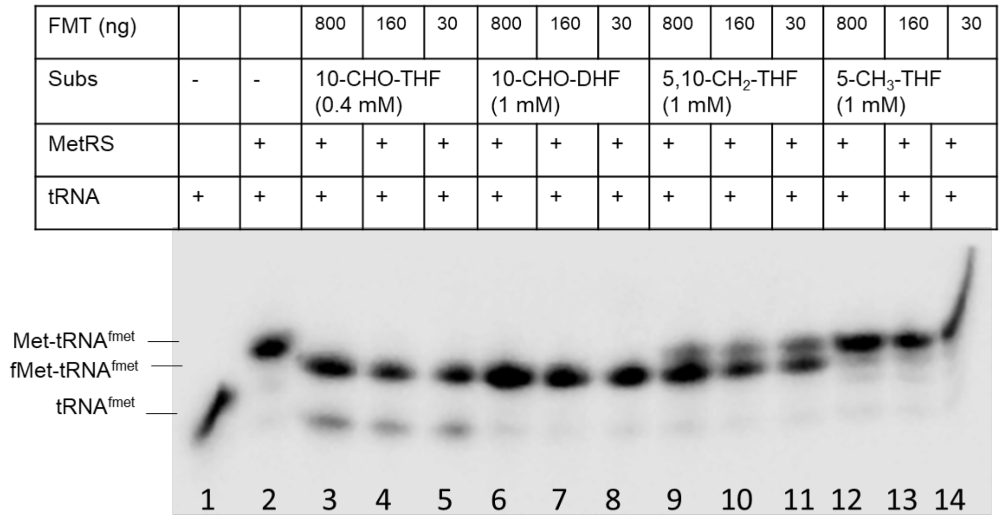

Figure S2. Formylation of Met-tRNA^{fMet} with 10-CHO-THF, 10-CHO-DHF and 5,10-CH_2-THF by Fmt. Total deacylated tRNA (tRNA^{fMet}) was converted into methionyl-initiator tRNA^{fMet} (Met-tRNA^{fMet}) with MetRS. The Met-tRNA^{fMet} was incubated with 10-CHO-THF, 10-CHO-DHF, 5,10-CH_2-THF or 5-CH_3-THF along with Fmt. Uncharged (tRNA^{fMet}), aminoacylated (Met-tRNA^{fMet}) and formylated (fMet-tRNA^{fMet}) forms of initiator tRNAs were separated on acid urea PAGE and analyzed by northern blotting with initiator-tRNA specific probe (met33). The tRNA^{fMet} and Met-tRNA^{fMet} and are shown as a control (Lane 1 and 2). The Met-tRNA^{fMet} was formylated with 10-CHO-THF (lane 3-5), 10-CHO-DHF (lane 6-8) and the *commercially* available 5,10-CH_2-THF (lane 9-11) but not with freshly synthesized 5,10-CH_2-THF (see Fig. 3, main text) or 5-CH_3-THF (lane 12 to 14).

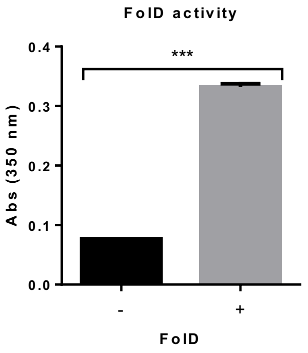

Figure S3. *In vitro* synthesis of 5,10-CH_2-THF by GlyA was confirmed by FolD. The 5,10-CH_2-THF was prepared enzymatically using GlyA from THF. The confirmation of 5,10-CH_2-THF synthesis was done by using FolD enzyme and $NADP^+$. The 5,10-CH_2-THF was converted to 5,10-CH^+-THF by FolD enzyme in the presence of $NADP^+$ which was measured by increase in the absorbance of the product, 5,10-CH^+-THF (λ_{max} = 350 nm, acidified pH). Error bars represent SD of two replicates. The p value calculated with unpaired-t-test was significant (p value = 0.0002).

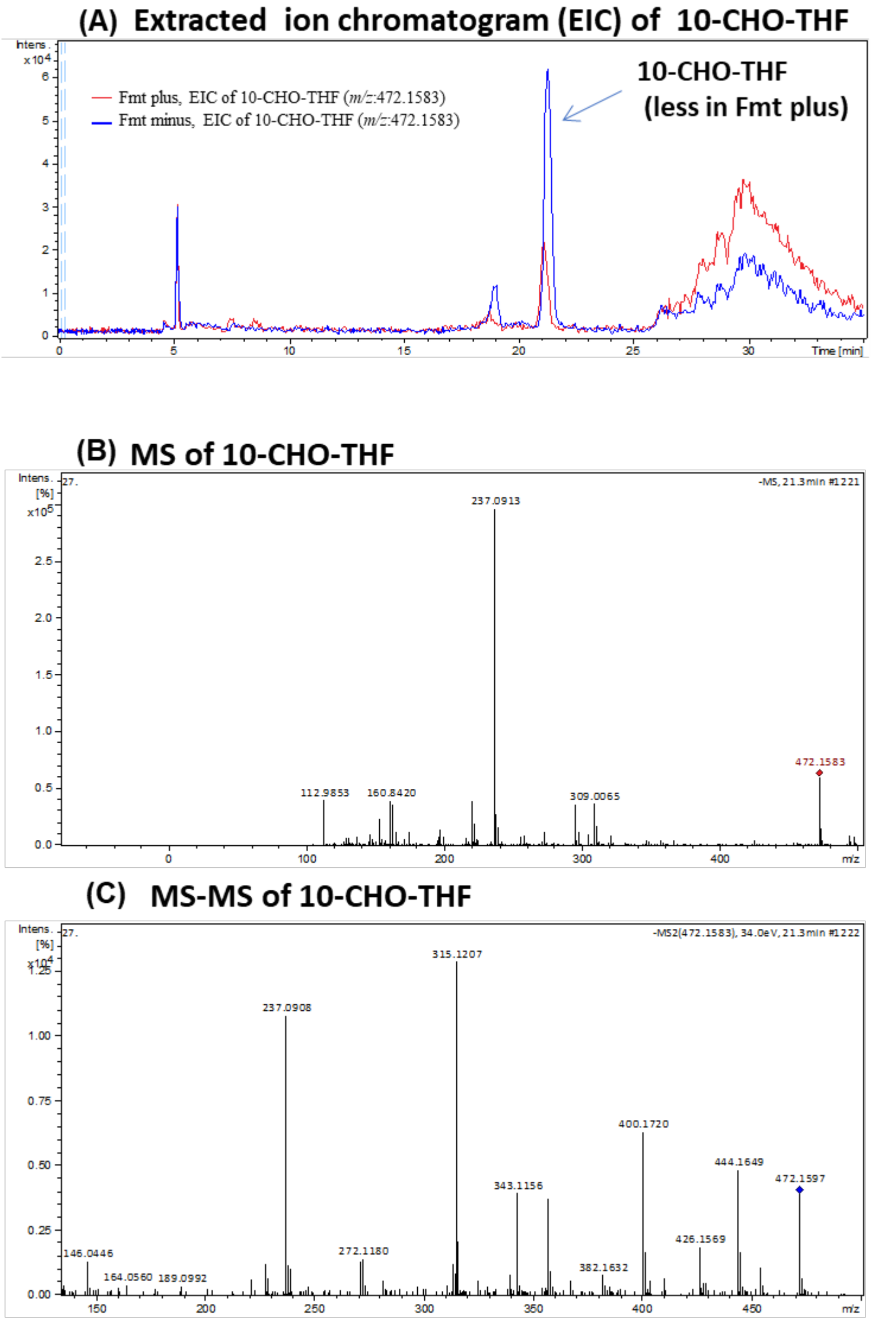

Figure S4. Identification of 10-CHO-THF as substrate of Fmt by LC-MS/MS. (A) The EIC intensity of 10-CHO-THF in the reaction mixture decreased after addition of Fmt (red line) in comparison to the one without Fmt (blue line). **(B)** The substrate, 10-CHO-THF was identified based on MS peak at 472.1583 (m/z). **(C)** MS ion of 10-CHO-THF (m/z = 472.1583) was further fragmented into many peaks by MS/MS analysis.

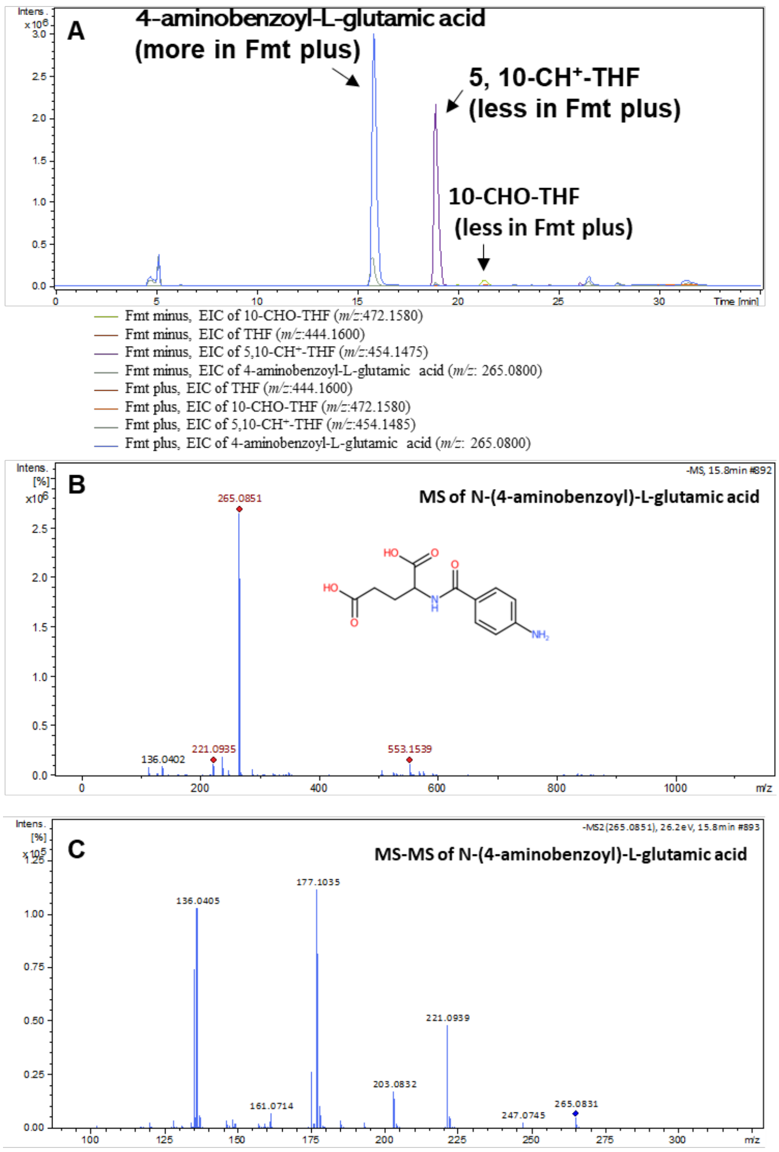

Figure S5. Identification of 4-aminobenzoyl-L-glutamic acid as the degradation product of THF by LC-MS/MS. (A) The EIC intensity of 5,10-CH^+-THF (*m/z*: 454.1475) in the reaction mixture decreased after addition of Fmt (grey line) in comparison to the one without Fmt (light brown line). The EIC intensity of 10-CHO-THF (*m/z*: 472.1580) in the reaction mixture decreased after addition of Fmt (red line) in comparison to the one without Fmt (light green line). The EIC intensity of 4-aminobenzoyl-L-glutamic acid (*m/z*: 265.080) in the reaction mixture increased after addition of Fmt (light blue line) in comparison to the one without Fmt (grey line). The EIC intensity of THF (*m/z*: 444.1600) in the reaction mixture is not visible (light brown line). **(B)** The degraded product of THF, 4-aminobenzoyl-L-glutamic acid was identified based on MS peak at 265.080 (*m/z*). **(C)** MS ion of 4-aminobenzoyl-L-glutamic acid (*m/z*: 265.080) was further fragmented into many peaks by MS/MS analysis.

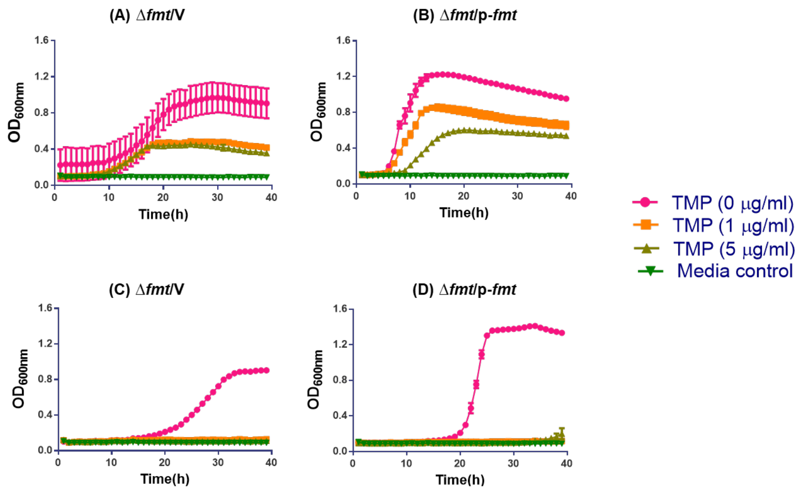

S6. Fmt overexpression causes slight toxicity and sensitivity to trimethoprim. *E. coli* Δ*fmt* harbouring plasmid alone (pACDH) or plasmid borne *fmt* (*p-fmt*, Tet^R) were grown in LB (**A and B**) and M9 media supplemented with thymine (50 µg/ml), adenine, glycine and methionine (100 µg/ml each) (**C and D**) at 37 ºC. The details of the strains are Δ*fmt*/V: Δ*fmt*/pACDH, Δ*fmt*/p-*fmt*: Δ*fmt*/pACDH-*fmt*.

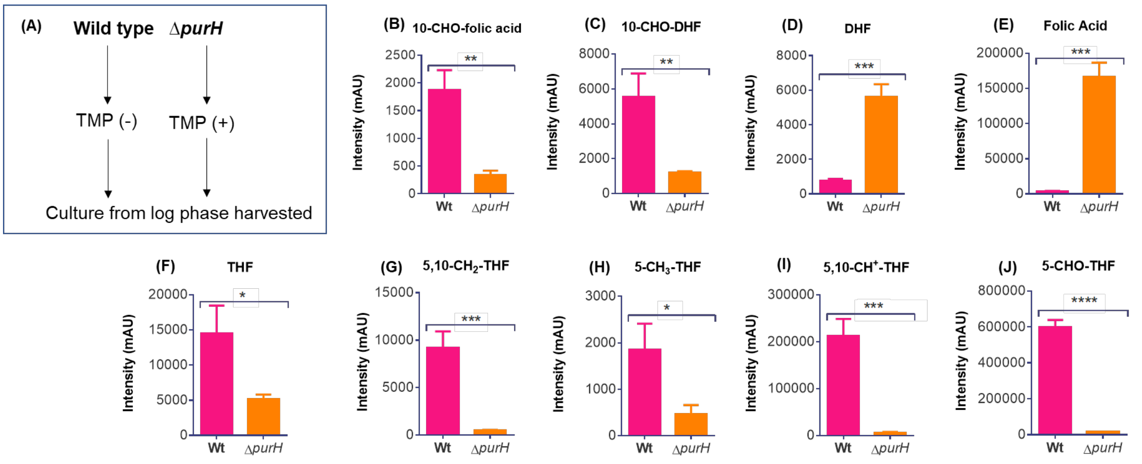

Figure S7. Analysis of folate metabolites from the log phase cultures by using LC-MS/MS. (**A**) Folate metabolites were analysed from wild type (BW25113) and Δ*purH* (treated with TMP) strains. (**B, C, F-J**) The intensity of 10-CHO-folic acid, 10-CHO-DHF, THF, 5,10-CH_2-THF, 5-CH_3-THF, 5,10-CH^+-THF and 5-CHO-THF (most likely) / 10-CHO-THF was decreased in the Δ*purH* strain treated with TMP compared to the wild type cell. (**D and E**) However, the intensity of DHF and folic acid were increased in the Δ*purH* strain treated with TMP compared to the wild type cells. Error bars represent SD of three replicates. The statistical significance was determined with unpaired two tailed t-tests. The *p* value calculated for the significant difference was < 0.05.

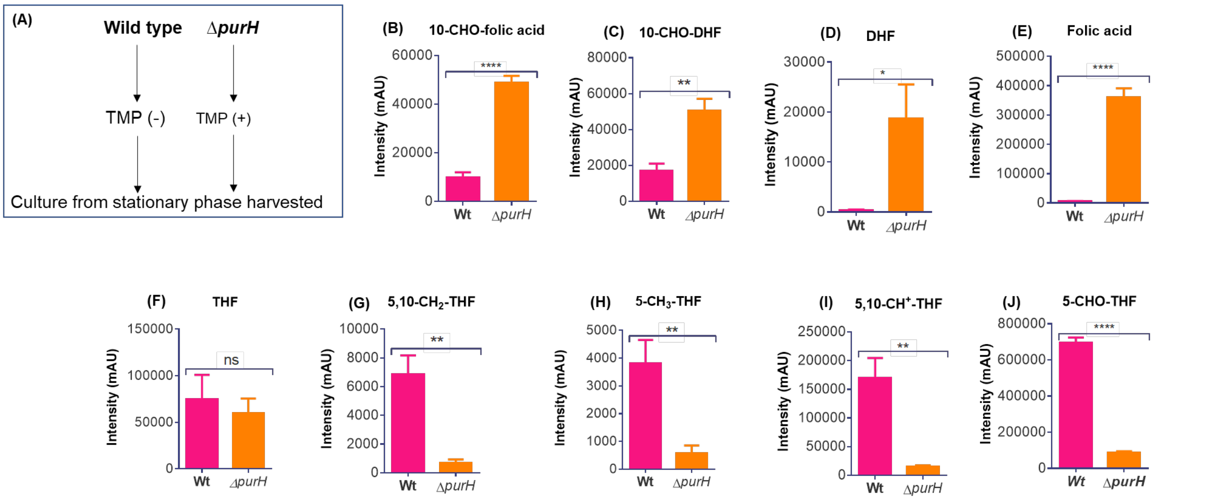

Figure S8. Analysis of folate metabolites from the stationary phase cultures using LC-MS/MS. (A) Folate metabolites were analysed from wild type (BW25113) and Δ*purH* (treated with TMP) strains. (**B-E**) The intensity of 10-CHO-folic acid, 10-CHO-DHF, DHF and folic acid were increased in the Δ*purH* strain treated with TMP compared to the wild type cells. (**F-J**) However, the intensity of THF, 5,10-CH_2-THF, 5-CH_3-THF, 5,10-CH^+-THF and 5-CHO-THF (most likely) / 10-CHO-THF was decreased in the Δ*purH* strain treated with TMP compared to the wild type cells. Error bars represent SD of three replicates. The statistical significance was determined with unpaired two tailed t-tests. The *p* value calculated for the significant difference was < 0.05.

Data S1

Identifier	HMDB01354
SMILES	C1C2CN(C=[N+]2C3=C(N1)NC(=NC3=O)N)C4=CC=C(C=C4)C(=O)NC(CCC(=O)O)C(=O)[O-]
MonoisotopicMass	455.155
InChI	InChI=1S/C20H21N7O6/c21-20-24-16-15(18(31)25-20)27-9-26(8-12(27)7-22-16)11-3-1-10(2-4-11)17(30)23-13(19(32)33)5-6-14(28)29/h1-4,9,12-13H,5-8H2,(H6-,21,22,23,24,25,28,29,30,31,32,33)/t12-,13+/m1/s1
MetFrag	395.25747209025343
CompoundName	5,10-Methenyltetrahydrofolic acid
MolecularFormula	C20H21N7O6
Identifier	HMDB01354

Precursor

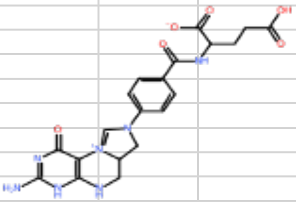

Fragments

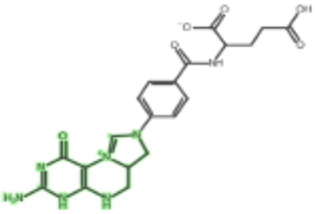

**Fragment 1**	
**Formula**	[C8H10N6O]-H-
**Mass**	205.0844
**Peak m/z**	205.0839

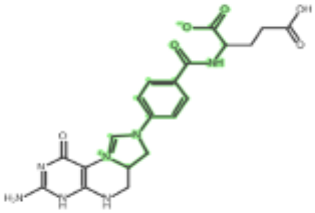

**Fragment 2**	
**Formula**	[C12H10N3O3+2H]-
**Mass**	246.08845
**Peak m/z**	246.0879

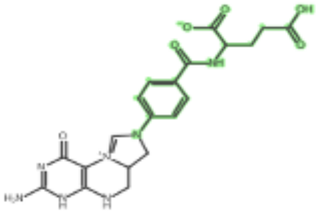

**Fragment 3**	
**Formula**	[C12H11N2O5+H]-
**Mass**	264.0752
**Peak m/z**	264.0894

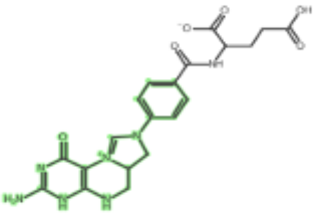

**Fragment 4**	
**Formula**	[C14H14N6O]-H-
**Mass**	281.11567
**Peak m/z**	281.1152

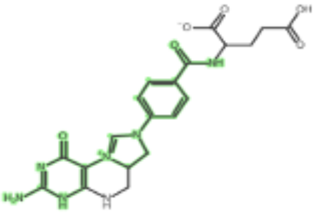

**Fragment 5**	
**Formula**	[C14H12N6O2]-
**Mass**	296.10275
**Peak m/z**	296.0896

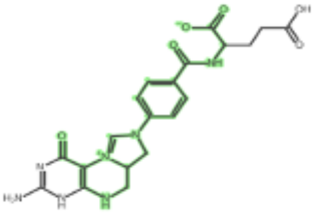

**Fragment 6**	
**Formula**	[C16H13N4O4]-
**Mass**	325.09426
**Peak m/z**	325.1056

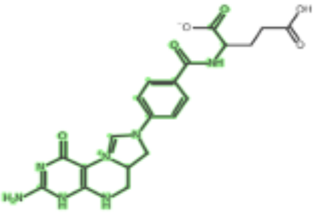

**Fragment 7**	
**Formula**	[C17H16N7O3+H]-
**Mass**	367.13988
**Peak m/z**	367.1517

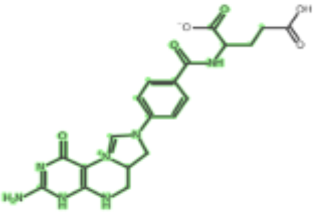

**Fragment 8**	
**Formula**	[C19H20N7O3-H]-H-
**Mass**	392.14771
**Peak m/z**	392.1472

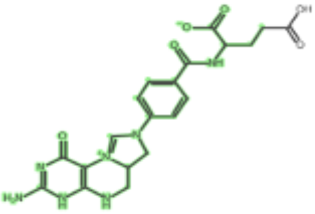

**Fragment 9**	
**Formula**	[C19H20N7O4]-
**Mass**	410.15828
**Peak m/z**	410.1572

Data S2

Identifier	HMDB01846
SMILES	C1C(NC2=C(N1)NC(=NC2=O)N)CNC3=CC=C(C=C3)C(=O)NC(CCC(=O)O)C(=O)O
MonoisotopicMass	445.171
InChI	InChI=1S/C19H23N7O6/c20-19-25-15-14(17(30)26-19)23-11(8-22-15)7-21-10-3-1-9(2-4-10)16(29)24-12(18(31)32)5-6-13(27)28/h1-4,11-12,21,23H,5-8H2,(H,24,29)(H,27,28)(H,31,32)(H4,20,22,25,26,30)/t11-,12?/m0/s1
MetFrag	460.1543571950054
CompoundName	Tetrahydrofolic acid
MolecularFormula	C19H23N7O6
Identifier	HMDB01846

Precursor

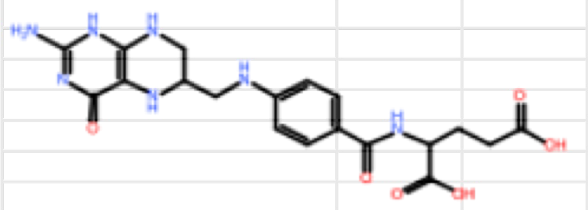

Fragments

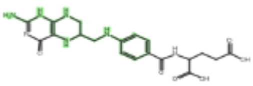

Fragment 1

Formula	[C12H15N5]-H-
Mass	228.12552
Peak m/z	228.1246

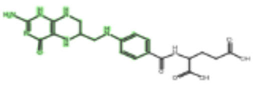

Fragment 2

Formula	[C13H15N6O]-
Mass	271.13133
Peak m/z	271.1305

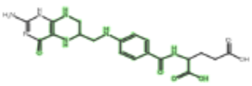

Fragment 3

Formula	[C15H15N4O4]-
Mass	315.10992
Peak m/z	315.12

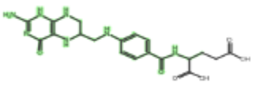

Fragment 4

Formula	[C16H19N7O2-H]-H-
Mass	339.14497
Peak m/z	339.1574

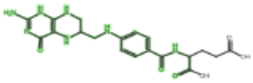

Fragment 5

Formula	[C16H17N7O3+2H]-
Mass	357.15554
Peak m/z	357.1672

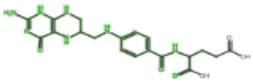

Fragment 6

Formula	[C18H21N7O3]-H-
Mass	382.16337
Peak m/z	382.1608

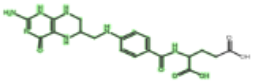

Fragment 7

Formula	[C18H22N7O4]-
Mass	400.17394
Peak m/z	400.1725

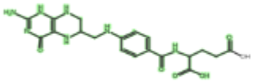

Fragment 8

Formula	[C19H22N7O5-H]-H-
Mass	426.15319
Peak m/z	426.153

Data S3

Identifier	HMDB06485
SMILES	C1C(=NC2=C(N1)NC(=NC2=O)N)CN(C=O)C3=CC=C(C=C3)C(=O)NC(CCC(=O)O)C(=O)O
MonoisotopicMass	471.15
InChI	InChI=1S/C20H21N7O7/c21-20-25-16-15(18(32)26-20)23-11(7-22-16)8-27(9-28)12-3-1-10(2-4-12)17(31)24-13(19(33)34)5-6-14(29)30/h1-4,9,13H,5-8H2,(H,24,31)(H,29,30)(H,33,34)(H4,21,22,25,26,32)/t13-/m0/s1
MetFrag	536.2417433555138
CompoundName	10-Formyldihydrofolate
MolecularFormula	C20H21N7O7
Identifier	HMDB06485

Precursor

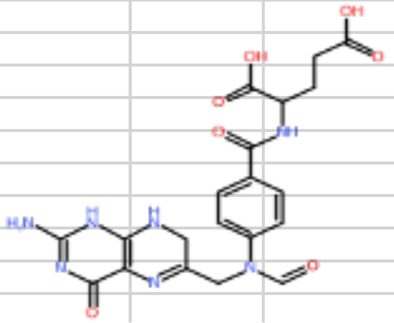

Fragments

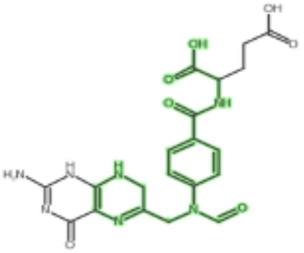

**Fragment 1**	
**Formula**	[C15H13N4O4]-
**Mass**	313.09426
**Peak m/z**	313.108

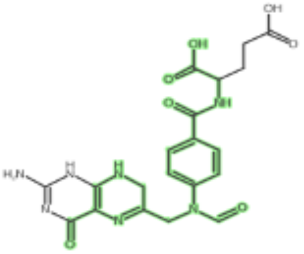

**Fragment 2**	
**Formula**	[C16H13N4O5]-
**Mass**	341.08917
**Peak m/z**	341.1029

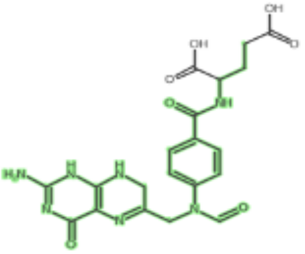

**Fragment 3**	
**Formula**	[C18H19N7O3]-H-
**Mass**	380.14771
**Peak m/z**	380.1478

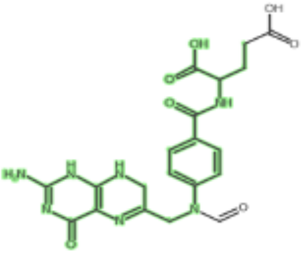

**Fragment 4**	
**Formula**	[C18H19N7O4+H]-
**Mass**	398.15828
**Peak m/z**	398.1669

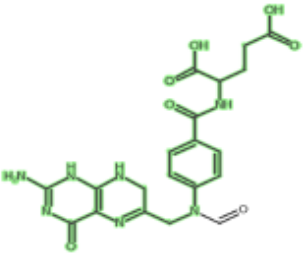

**Fragment 5**	
**Formula**	[C19H20N7O6]-
**Mass**	442.1481
**Peak m/z**	442.144

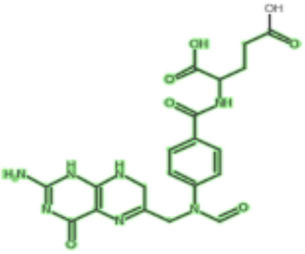

**Fragment 6**	
**Formula**	[C20H20N7O6-H]-H-
**Mass**	452.13244
**Peak m/z**	452.1325

Data S4

Identifier	HMDB01056
SMILES	C1C(=NC2=C(N1)NC(=NC2=O)N)CNC3=CC=C(C=C3)C(=O)NC(CCC(=O)O)C(=O)O
MonoisotopicMass	443.155
InChI	InChI=1S/C19H21N7O6/c20-19-25-15-14(17(30)26-19)23-11(8-22-15)7-21-10-3-1-9(2-4-10)16(29)24-12(18(31)32)5-6-13(27)28/h1-4,12,21H,5-8H2,(H,24,29)(H,27,28)(H,31,32)(H4,20,22,25,26,30)/t12-/m0/s1
MetFrag	112.24120972774742
CompoundName	Dihydrofolic acid
MolecularFormula	C19H21N7O6
Identifier	HMDB01056

Precursor

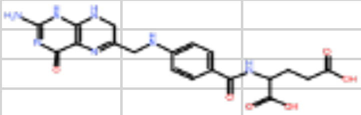

Fragments

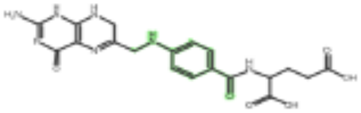

**Fragment 1**	
**Formula**	[C8H7NO]-
**Mass**	133.05334
**Peak m/z**	133.053

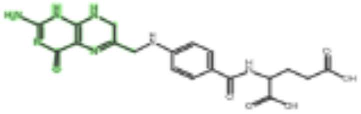

**Fragment 2**	
**Formula**	[C7H8N5O-H]-H-
**Mass**	176.05779
**Peak m/z**	176.0575

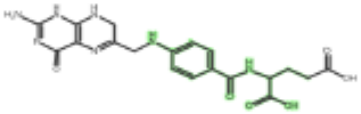

**Fragment 3**	
**Formula**	[C11H12N2O3+H]-
**Mass**	221.09321
**Peak m/z**	221.0925

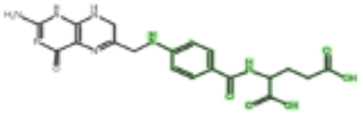

**Fragment 4**	
**Formula**	[C12H13N2O5]-
**Mass**	265.08303
**Peak m/z**	265.0841
